# Interpreting the Climatic Effects on Xylem Functional Traits in Two Mediterranean Oak Species: The Role of Extreme Climatic Events

**DOI:** 10.3389/fpls.2016.01126

**Published:** 2016-08-02

**Authors:** Angelo Rita, Marco Borghetti, Luigi Todaro, Antonio Saracino

**Affiliations:** ^1^Dipartimento di Agraria, Università di Napoli Federico IIPortici, Italy; ^2^Scuola di Scienze Agrarie, Forestali, Alimentari ed Ambientali, Università della BasilicataPotenza, Italy

**Keywords:** tree-ring, hydraulic conductivity, quantitative wood anatomy, Mediterranean climate, pointer years

## Abstract

In the Mediterranean region, the widely predicted rise in temperature, change in the precipitation pattern, and increase in the frequency of extreme climatic events are expected to alter the shape of ecological communities and to affect plant physiological processes that regulate ecosystem functioning. Although change in the mean values are important, there is increasing evidence that plant distribution, survival, and productivity respond to extremes rather than to the average climatic condition. The present study aims to assess the effects of both mean and extreme climatic conditions on radial growth and functional anatomical traits using long-term tree-ring time series of two co-existing *Quercus* spp. from a drought-prone site in Southern Italy. In particular, this is the first attempt to apply the Generalized Additive Model for Location, Scale, and Shape (GAMLSS) technique and Bayesian modeling procedures to xylem traits data set, with the aim of (i) detecting non-linear long-term responses to climate and (ii) exploring relationships between climate extreme and xylem traits variability in terms of probability of occurrence. This study demonstrates the usefulness of long-term xylem trait chronologies as records of environmental conditions at annual resolution. Statistical analyses revealed that most of the variability in tree-ring width and specific hydraulic conductivity might be explained by cambial age. Additionally, results highlighted appreciable relationships between xylem traits and climate variability more than tree-ring width, supporting also the evidence that the plant hydraulic traits are closely linked to local climate extremes rather than average climatic conditions. We reported that the probability of extreme departure in specific hydraulic conductivity (Ks) rises at extreme values of Standardized Precipitation Index (SPI). Therefore, changing frequency or intensity of extreme events might overcome the adaptive limits of vascular transport, resulting in substantial reduction of hydraulic functionality and, hence increased incidence of xylem dysfunctions.

## Introduction

An accepted picture for the Mediterranean region is the ongoing trend toward higher temperature and reduced precipitation, associated to an increase in the frequency and magnitude of climatic extremes, which are expected to have detrimental effects on trees and forest biomes (Easterling et al., 2000; IPCC, 2013). Drought imposed by changes in rainfall patterns and temperature anomalies were implicated in a number of well-documented drought-induced tree mortalities and forest decline episodes, with likely consequences on species distribution and community structure (e.g., Allen et al., 2010; Anderegg et al., 2012).

Interestingly, extreme climatic events are increasingly considered to play a major role in tree mortality, and variation in xylem anatomical traits linked to tree hydraulic properties has received considerable attention in recent decades as an important plant acclimation process (for a review on this issue, see Jentsch et al., 2007). Specifically, both observational and experimental studies to date reported that variability and extremes in climate are more important drivers of ecosystem processes than mean conditions (Royer et al., 2011; Smith, 2011; Thompson et al., 2013). For example, a number of studies that experimentally imposed climate extremes via field experiments clearly described the negative impact of extreme drought on the xylem hydraulic function and productivity (Jentsch et al., 2011; Barigah et al., 2013; Urli et al., 2013). However, there are so far at least three factors limiting our understanding of the impacts of extreme events on plant hydraulic functionality: (i) the frequently loose definition of extreme events, which needs refinement because climate change involves modifications in both mean and variability (Smith, 2011; Lloret et al., 2012); (ii) the difficult comparison between case studies, particularly due to their heterogeneity in temporal and spatial scales and the disparity of response variables (Reyer et al., 2013); (iii) still incomplete information on the phenotypic plasticity of trees, that is, the potential to modify their form and function in response to environmental changes, especially to changing climate (Fonti et al., 2010). Phenotypic plasticity can reduce mortality risk when plants are exposed to new conditions. The most common plastic responses of trees to drought are a reduction in leaf area to restrict water loss (DeLucia et al., 2000) and increased root growth to enhance access to water and nutrients. In turn, the structure and hydraulic function of xylem can also vary within a single species in response to climate conditions. For instance, variation in tree-ring widths, vessel diameters, or distribution has often been used to reconstruct information about past environmental conditions and infer the hydraulic function of xylem (see Fonti et al., 2010 for a review). Nevertheless, acclimation is not instantaneous, and it is sensitive to several factors. For example, full acclimation to a temperature shift may take between a few days to weeks and might be further affected by interactions with other factors such as drought (Valladares et al., 2007). Therefore, xylem plastic adjustments may not be able to cope with the effect of rapid and extreme climatic events.

Nevertheless, for high temporal resolution, long-term xylem traits chronologies have shown to be sensitive indicators of climate variability. Promising results have been obtained from studies on water conducting elements across a range of hardwoods species (e.g., Maherali et al., 2004). In particular, several authors successfully revealed a clear signal in vessel traits of ring-porous species mainly linked to the water availability (Fonti and García-González, 2004; Campelo et al., 2010; Gea-Izquierdo et al., 2012); for sub-Mediterranean oaks it was demonstrated that most of the variability in early wood vessel size could be explained by spring precipitations (García-González and Eckstein, 2003; González-González et al., 2014). Moreover, several studies recently acknowledged the importance of the ontogenetic changes on the hydraulic design of woody plants (Olson et al., 2014), suggesting careful evaluation of the climatic information from tree-ring time series (Carrer et al., 2015).

The present study aims to disentangle the effects of mean and extreme climatic variability on functional anatomical vessel traits from long-term tree-ring series. Specifically, we hypothesized that (i) vessel traits may reflect more substantially the climatic signal than tree-ring width, (ii) a closely link between extreme values in tree-ring series (both ring with and specific hydraulic conductivity) and site-specific extreme climatic condition occurs.

We used *Quercus cerris* L. and *Quercus pubescens* Willd. as model species, both are ring-porous species with large diameter early-wood vessels that allow water movement with a minimum of hydraulic resistance (Tyree and Zimmermann, 2002). However, experimental evidences indicates higher cavitation rates and vulnerability to embolism in the former species (Borghetti et al., 1993; Lo Gullo et al., 1995; Nardini et al., 1999), whose large vessels could enhance water transport efficiency but compromise the safety of the xylem (Tyree and Zimmermann, 2002). Recent studies have reported several oak-decline episodes in the Iberian Peninsula during the 1980s and 1990s when several intense summer droughts episodes occurred (Peñuelas et al., 2001; Corcuera et al., 2004). To this aim, the Generalized Additive Model for Location, Scale and Shape (GAMLSS, Rigby and Stasinopoulos, 2005) and a Bayesian logistic simulation were used to perform a high-resolution examination of tree-ring traits and climate relationships. The ability of the aforementioned tools to handle non-linear data structures can better represent the complex relationship between xylem functional traits and environmental variables.

## Materials and methods

### Study site and plant material

The study was carried out on trees sampled in a mountain forest in the Pollino National Park in Southern Italy, close to the Mediterranean coast. The climate is influenced by differences in altitude, slopes, and proximity to the sea. There is a typical Mediterranean seasonal alternation between dry and warm summers and rainy winters.

Temperatures were collected from Castrovillari (39° 83′ N, 16°19′ E, 343 m a.s.l.) and precipitation data from San Lorenzo Bellizzi (39° 88′ N, 16° 32′ E, 851 m a.s.l.) meteorological stations (Italian Hydrographic Service, SIMI). Temperatures were corrected for altitude by applying a coefficient of −0.007°C m^−1^ (ICAO, 2002). The average annual precipitation is 1065 mm distributed as 39.5% in winter, 23.7% in spring, 29.2% in autumn, and 7.6% in summer. The Mediterranean sub-humid climate is characterized by warm summers (at this elevation, 18.06°C is the average temperature for July through August) and cold winters (average 1.8°C for December through February). Mean annual temperature is 9.4°C and snowfalls are generally distributed from November to April.

In recent decades, a maximum of 120 days of dry weather was recorded in summer.

At the study site (39°56′58.8″N, 16°10′32.4″E, elevation 1050 m a.s.l.) the forest consist of scattered trees with a canopy height of ~20 m, with few trees reaching a tree height of ~28 m.

Two 5 mm diameter cores from each of 16 *Q. cerris* and 15 *Q. pubescens* tall adult trees with a diameter at breast height (DBH) >40 cm were collected with an increment borer for tree-ring analysis. For each core sample, tree-rings were first visually cross dated (Yamaguchi, 1991) and then measured to the nearest 0.01 mm using the incremental measuring table SMIL3 (Corona et al., 1989) interfaced with data acquisition software. Finally, the COFECHA software (Holmes, 1983) was used to check for the presence of cross-dating errors and the expressed population signal (EPS) was calculated with the package “*dplR*” (Bunn, 2008) in the R statistical suite (R Core Team, 2015) to quantify the common variability among tree-ring series. An average tree-ring chronology spanning from 1926 to 2012 was obtained; the EPS value exceeded the suggested threshold 0.85 level (EPS = 0.92), indicating a high degree of common variability between tree-ring series (Wigley et al., 1984).

### Xylem anatomy and specific hydraulic conductivity

A subsample of 10 cross-dated cores from the two species was investigated for xylem anatomical characteristics and xylem hydraulic conductivity after checking the presence of reaction wood or wounding (Arbellay et al., 2012).

A sliding microtome (HM 400, Microm International GmbH, Walldorf, Germany) was used to obtain 20 μm thick transverse sections from split micro-sections of entire wood cores (from 1 to 1.5 cm long). Histological preparations were then obtained by staining sections with 2% astrablue and 1% safranin solutions, which resulted in unlignified cells appearing blue and lignified cells appearing red (Schweingruber and Poschlod, 2005). Sections were subsequently dehydrated using a series of ethanol solutions of increasing concentrations, washed with xylol, and embedded in Canada balsam.

Annual ring images from transverse sections were captured with a CCD digital camera (Skopkam DCM300) mounted on a reflected light microscope (AxioPhot, Carl Zeiss, Jena, Germany). Sequential images were subsequently stitched using the Microsoft Image Composite Editor (ICE 1.3.5), and analyzed with the image-analysis software ImageJ v.1.40 (National Institute of Health, Bethesda, MD, USA, http://rsb.info.nih.gov/ij). Images were first converted from 24-bit color into 8-bit grayscale and then the objects contour was produced in a threshold binary image (mask) in which only the particles of interest were retained, in our case the vessels lumen. Before any measuring, the image was calibrated from a scale bar of known length in the image. The particle analysis function led us to calculate for each tree-ring, in a chosen surface (S_Xylem_ = W_r_^*^l, where W_r_ is ring width and l = 2 mm) bounded by rays, the vessel number (N), the vessel lumen area (A), and the Cartesian coordinates of each vessel (>480 μm^2^). Careful visual inspection was also performed to verify all vessel elements and non-vascular elements included.

Since vessels are not exactly circular but mostly elliptical, the diameter of each vessel was calculated as:

$$\begin{array}{l} \text{d}=\;{\left(\frac{32{\left(\text{ab}\right)}^{3}}{{\text{a}}^{2}{\text{b}}^{2}}\right)}^{\frac{1}{4}} \end{array}$$

where a and b are major and minor perpendicular lumen diameters, respectively (Lewis, 1992).

Based on the vessel contribution to hydraulic conductance, we calculated the hydraulically weighted mean diameter (D_h_) for each ring according to Tyree and Zimmermann (2002):

$$\begin{array}{l} {\text{D}}_{\text{h}}={\left(\frac{1}{\text{n}}\sum_{1}^{\text{n}}{\text{d}}^{4}\right)}^{\frac{1}{4}} \end{array}$$

According to the Hagen–Poiseuille equation, theoretical hydraulic conductivity (K_h_, m^4^ MPa^−1^ s^−1^) was calculated from the vessel radii (r) as

$$\begin{array}{l} {\text{K}}_{\text{h}}=\frac{\pi \sum_{1}^{\text{n}}{\text{r}}^{4}}{8\eta } \end{array}$$

where η is the viscosity of water at 20°C (1.002 10^−3^ Pa s).

The tree-ring specific hydraulic conductivity (K_s_, kg m^−1^ MPa^−1^ s^−1^) was estimated by dividing the theoretical hydraulic conductivity (K_h_) by the tree-ring surface area (S_i_) and multiplying with the density of water (ρ) at 20°C (998.20 kg m^−3^), according to the modified Hagen-Poiseuille equation reported by Tyree and Ewers (1991)

$$\begin{array}{l} {\text{K}}_{\text{s}}=\frac{{\text{K}}_{\text{h}}\rho }{{\text{S}}_{\text{Xylem}}} \end{array}$$

Average vessel size (A_av_) and vessel density (d_v_), determined as the ratio between the number of vessels and the area analyzed, were also calculated.

### Data analysis

Statistical analyses were performed for the period 1952–2012 (60 years). To explore the relationships between xylem traits (W_r_ and K_s_) and climatic variables (temperature and precipitation) we applied Generalized Additive Models for Location, Scale, and Shape (GAMLSS) proposed by Rigby and Stasinopoulos (2005) as semiparametric regression model. GAMLSS overcomes some limitations associated with Generalized Linear Models (GLMs) and GAMs by providing a flexible modeling framework that allows the use of more general distributions, such as highly skewed or kurtotic distributions, which may be more appropriate for modeling the record of interest. The number of parameters represented in the GAMLSS distributions varies from one to four, with almost all distributions represented by a location (μ) and scale (σ) parameter and some distributions represented by one or two shape parameters (υ and τ) to represent skewness and kurtosis in the response variable data. For this reason, the form of the distribution assumed for the response variable is y~f(x| μ, σ, υ, τ). Computational implementation was performed using the package “*gamlss*” (Stasinopoulos and Rigby, 2007) in the R statistical suite (R Core Team, 2015). The model also included the cambial age as covariate and a random intercept term to account for variation among trees. The resulting estimations, are based on a P-Spline (*ps*, Penalised B-spline) smoothing function (Eilers and Marx, 1996), where the smoothing parameters (and hence the effective degrees of freedom) are estimated using the local maximum likelihood method.

The model building process consisted in comparing many competing models for which different combinations of components (i.e., *M*_model_ = *D*_distribution_, *G*_link function_, *T*_predictors_, λ_smoothing_) were tried. Minimizing the Akaike Information Criterion (AIC) was used for the model selection (Akaike, 1998). Selected models were checked for the independence and normality of the residuals by worm plots and qq-plots (Buuren, 2007).

In addition, we used the LMS (λ-μ-σ) method (Cole and Green, 1992) to determine the age-specific trends in the xylem traits, thereby allowing examination of the temporal trends in specific percentile points of W_r_ and K_s_. They were estimated via *lms*() in the “*gamlss*” R package, where the first three moments of the measurement frequency distribution were modeled as cubic smoothing spline curves, based on Box Cox-type transformations of data. The LMS method implicitly leads to non-crossing curves via a scaling function.

Given that low summer precipitation concomitantly with high temperatures were found to be the most limiting factor for the development of the Mediterranean tree species, we tested the hypothesis of an influence of climate on extreme deviation in the ring width (W_r_) and specific hydraulic conductivity (K_s_), by applying one of the most commonly used procedures in classical dendrochronological studies: pointer years analysis. In fact, as stated by Schweingruber et al. (1990), event and pointer years are a suitable proxy of the extreme climatic events to which trees have been exposed in the past. We calculated the pointer years on the individual series of W_r_ and K_s_ with the “*pointRes*” (van der Maaten-Theunissen et al., 2015) R package by using the normalization in a moving window (|W| = 5 years) according to Cropper (1979; *cf*. Schweingruber et al., 1990). Pointer years were defined as those years with absolute values above a threshold of 0.75 according to Cropper (1979). This method z-transforms tree growth in year *i* within a symmetric moving window of *n* years, thereby providing the number of standard deviations that tree growth deviates in individual years from the window average. Subsequently, positive and negative pointer years were represented by dichotomous variables coded as 0 and 1, respectively.

Hence, a Bayesian logistic regression was adopted to test the link between pointer years (where pointer years are *Bernoulli* distributed) and climate through JAGS + “*rjags*” R package cross-platform Plummer (2003). The predictor variable comprised the Standardized Precipitation Index (SPI) computed through the “SPEI” R package (Beguería and Vicente-Serrano, 2013).

Then, with W_r_ and K_s_ pointer years values (Y) in {0, 1} the estimated model was:

$$\begin{array}{l} \text{Y}\sim Bernoulli\;\left(y_{\text{i}}|\pi_{\text{i}}\right)\\ \text{where},\pi_{i}\equiv \mathrm{Pr}\left(y_{i}\;=1|{SPI}_{i},\beta \right)=\frac{1}{1+\text{exp}\left(-{SPI}_{i}\beta \right)} \end{array}$$

JAGS used Markov Chain Monte Carlo (MCMC) to generate a sequence of dependent samples from the posterior distribution of the parameters by assuming a weakly informative prior distribution (0, 0.5), as proposed by Gelman et al. (2008). Simulation was performed by running four chains with 20,000 total iterations per chain and 10,000 initial samples “burn-in.” Convergence diagnostics (provided by the *coda* R package) were visually checked by the autocorrelation plot, Geweke's diagnostic and the Gelman-Rubin shrink factor (Brooks and Gelman, 1998).

Then we modeled the change in the probability of presence (1) − absence (0) of Wr and Ks pointer years at the extreme upper and lower SPI event (exceeding the 90th and 10th percentile, respectively) by running 1000 bootstrap simulation of quantities of interest (QI) from the posterior density of the Bayesian model, as suggested by King and Wittenberg (2000).

## Results

**Figure 1:**
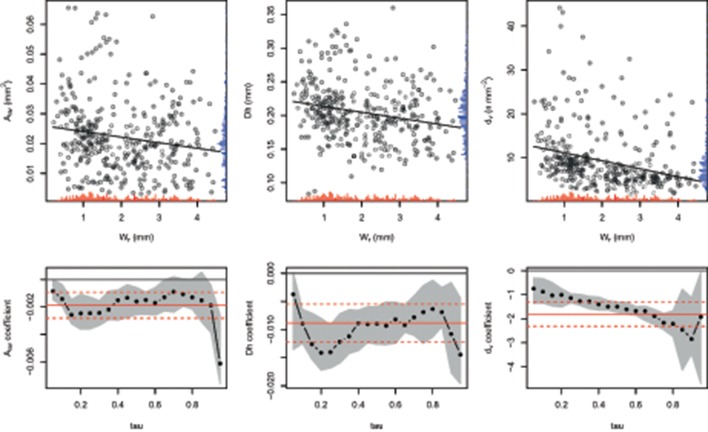
**Relationships between the average vessel size [A_**av**_; β = −0.002, ***F***_**(1, 375)**_ = 10.52, ***p*** < 0.01], hydraulic diameter [D_**h**_; β = −0.009, ***F***_**(1, 375)**_ = 18.77, ***p*** < 0.001], vessel density [d_**v**_; β = −1.808, ***F***_**(1, 375)**_ = 34.12, ***p*** < 0.001], and tree-ring width [W_**r**_] (upper panel)**. Red and blue rugs inside the plots show distribution of the *x* and *y* variables, respectively. Slope of the estimated linear quantile regression for the xylem traits as a function of the τth quantile τ **(lower panels)**. Full red lines are the least squares estimates for the coefficients and the red dashed lines are the 95% confidence intervals for the least squares estimates. The gray area represents the 95% confidence interval (1000 replicate bootstrap) for the quantile regression estimates (full circles, each 5th quantile).

Relationships between xylem traits and W_r_ showed that the standard linear regression fit to these data was significantly different from zero for A_av_, D_h_, and d_v_ (Figure 1, upper panels); in particular, all outcomes appeared to be negatively related to the tree-ring width. Moreover, the regression coefficient for all traits showed different significant patterns according to the quantile considered (Figure 1, lower panels).

**Figure 2:**
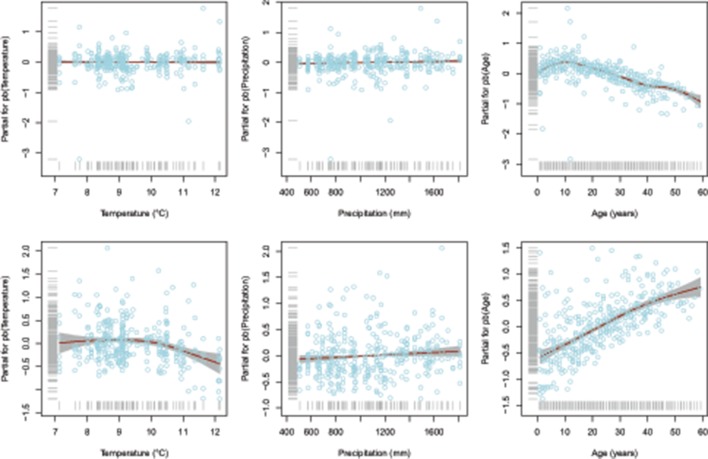
**Partial regression plots for fitted W_**r**_ (upper panels) and K_**s**_ (lower panels) models**. The solid line is modeled trends and the shaded area is 95% confidence intervals. Gray rugs on the y-axis and x-axis represent distribution of the partial residuals and covariates, respectively. Note free scales of partial residuals.

**Figure 3:**
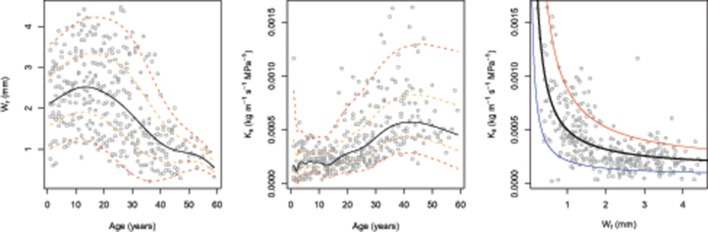
**Tree-ring width (W_**r**_) and specific hydraulic conductivity (K_**s**_) centile curve (left and central panels, respectively) using the LMS method as a function of cambial age**. Each panel shows the 5^th^, 25^th^, 50^th^, 75^th^, and 95^th^ percentile from the bottom to the top, respectively. Scatter plot between K_s_ and W_r_ with non-linear [K_s_ ~ I(1/W_r_^*^ a) + b] quantile regression line **(right panel)**. The black line is median (a, β = 3.505e-04, *p* < 0.001; b, β = 1.377e-04, *p* < 0.001), the red line is the 90th quantile (a, β = 0.00069, *p* < 0.001; b, β = 0.00018, *p* < 0.001), and the blue line is the 10th quantile (a, β = 0.00015, *p* < 0.001; b, β = 0.00008, *p* < 0.001).

Results of the fitted GAMLSS highlighted significant temperature, precipitation, and cambial age effect for K_s_, whilst only the age effect was depicted for W_r_ (Table 1). In particular, as expected, increase of both temperature and precipitation led to decrease and increase in the K_s_, respectively (Figure 2). Interestingly, both W_r_ and K_s_ models indicated that these variables are strongly dependent on cambial age. In this regard, further investigation (Figure 3) indicated an inverse trend across the overall age distribution and in all percentiles for W_r_ and K_s_, where the rate of increase in hydraulic conductivity was directly related to age. In particular, higher percentile levels were reached from 10 to 20 years for W_r_ and from 40 years onwards for K_s_. Moreover, there were larger increases in the upper than the lower percentiles in K_s_, particularly from age >20 years. The typical monotonic increasing trend of hydraulic conductivity tends to be low when choosing the 5th percentile, i.e., selecting only the smallest conduits per year.

**Table 1:** **Summary statistics for the best fitted GAMLSS for tree-ring width (W_**r**_) and specific hydraulic conductivity (K_**s**_) variables**.

		**W_r_**					**K_s_**				
		**Est**.	**Std. Error**	***t*-value**	**Pr(>|t|)**	***df***	**Est**.	**Std. Error**	***t*-value**	**Pr(>|t|)**	***df***
μ	Intercept	1.12E+00	6.54E-02	17.14	^***^	–	−8.04E+00	1.68E-01	−47.86	^***^	–
	Temperature	−3.48E-03	8.13E-03	−0.43	0.07	2.02	−7.75E-02	2.03E-02	−3.83	^***^	4.25
	Precipitation	3.49E-06	2.62E-05	2.42	0.17	2	8.78E-05	6.37E-05	1.38	^*^	2.03
	Age	−1.93E-02	6.24E-04	−30.91	^***^	8.86	2.64E-02	1.43E-03	18.45	^***^	3.42
σ	Intercept	2.55E-01	3.13E-01	0.82	0.41	–	−4.59E-01	3.03E-01	−1.51	0.13	–
	Temperature	2.75E-02	3.60E-02	0.76	0.04	2	−1.52E-02	3.49E-02	−0.44	0.66	2.93
	Precipitation	−3.01E-04	1.20E-04	−2.45	^*^	2	−3.92E-06	1.19E-04	−0.03	0.97	2
	Age	−3.37E-02	2.60E-03	−12.60	^***^	10.31	−1.52E-02	2.59E-03	−5.86	^***^	3.46
υ	Intercept	1.34E+00	3.63E-01	3.69	^***^	–	−1.34E+00	7.66E-01	−1.74	0.08	–
	Temperature	−3.91E-01	3.58E-02	−10.91	^***^	2	1.37E-01	8.22E-02	1.67	0.10	2
	Precipitation	2.09E-03	1.45E-04	14.36	^***^	10.9	2.05E-04	3.11E-04	0.66	0.51	2
	Age	2.05E-03	4.36E-03	0.47	0.64	21.69	−3.03E-03	7.00E-03	−0.43	0.67	2
τ	Intercept	–	–	–	–	–	7.59E-01	1.21E-01	6.26	^***^	–
Family				BCCGo					BCPEo		
AIC				637					−5308		

Parameters of the distribution families are μ (mean, location parameter), σ (centile-based coefficient of variation, scale parameters), υ (skewness), and τ (kurtosis). Stars mean statistical significance for

*** *p < 0.001*,

* *p < 0.05*.

*Est, Estimate; BCCGo, Box-Cox-Cole-Green-orig.; BCPEo, Box-Cox Power Exponential-orig.; AIC, Akaike Information Criterion*.

**Figure 4:**
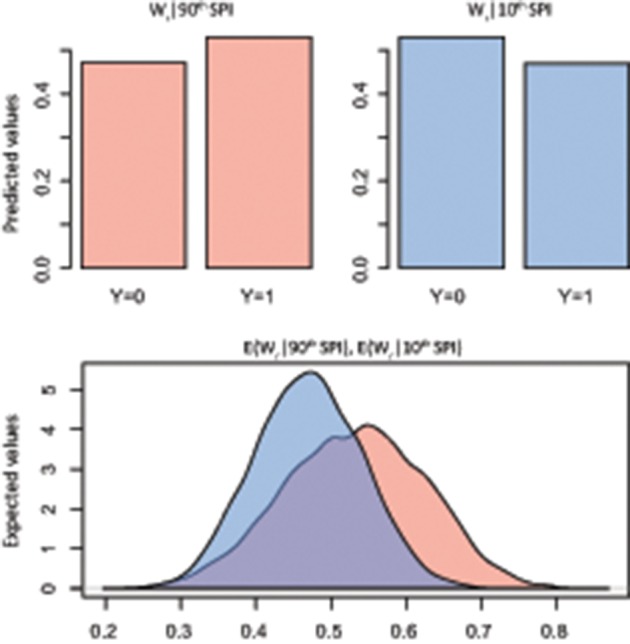
**Bayes simulation of quantities of interest**. Predicted probability (bar plot) and expected probability (density plot) of presence [1] − absence [0] of W_r_ pointer years (Y) at the 90^th^ (red) and 10^th^ (blue) percentile of the Standardized Precipitation Index (SPI). E(W_r_|90^th^ SPI), mean = 0.53(±0.09), 2.5% = 0.34, 97.5% = 0.70; E(W_r_|10^th^ SPI), mean = 0.46(±0.07), 2.5% = 0.33, 97.5% = 0.60.

**Figure 5:**
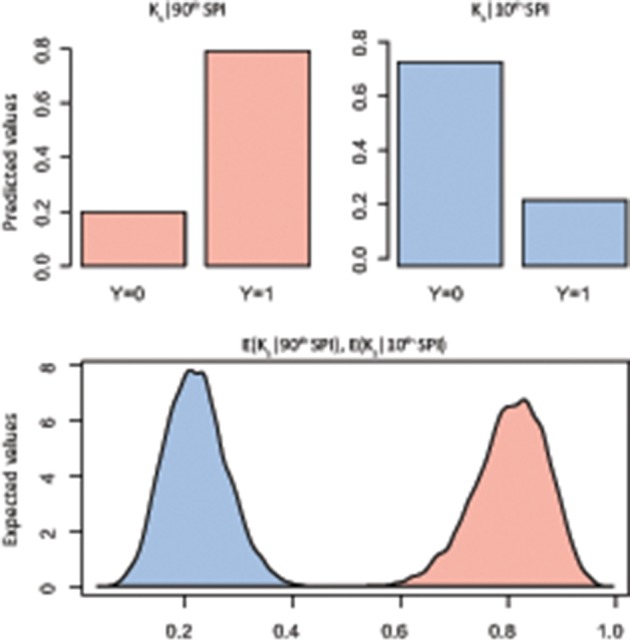
**Bayes simulation of quantities of interest**. Predicted probability (bar plot) and expected probability (density plot) of presence [1] − absence [0] of K_s_ pointer years (Y) at the 90^th^ (red) and 10^th^ (blue) percentile of the Standardized Precipitation Index (SPI). E(K_*s*_|90^th^ SPI), mean = 0.78(±0.06), 2.5% = 0.64, 97.5% = 0.89; E(K_s_|10^th^ SPI), mean = 0.24(±0.05), 2.5% = 0.14, 97.5% = 0.36.

The summary statistics table of the Bayesian logistic model (Table 2) showed the marginal posterior distribution for parameter α (intercept) and β_SPI_ (coefficient; see Supplementary Figures S1, S2 for trace and density plots). Interestingly, for K_s_ the 95% credible interval for β_SPI_ is positive, indicating with very high probability that the β term is positive: exposure to high SPI increases the probability of positive deviation in K_s_. No similar evidence was found for W_r_. However, simulation of quantities of interest, in terms of predicted probability of a success, was more informative than simply reporting the model estimates (Figures 4, 5). In particular, results of simulation reported the predicted and the expected probability (*sensu* King and Wittenberg, 2000) of the presence/absence of pointer years at the 90th and 10th percentile of the SPI with 95% confidence level. Simulation in Figure 4 confirms that there was no clear probability that formation of pointer years in W_r_ was affected by changes in SPI. Indeed, we reported that in spite of extreme values of SPI (above the 90th percentile), there is the 53% probability of occurrence of positive pointer years. On the other hand, SPI values below the 10th percentile are expected to affect the formation of a negative pointer year at 46% probability. However, contrasting results were found for simulation of K_s_ pointer years (Figure 5). We expected the 78% probability of occurrence of positive pointer years at the 90th percentile of SPI and only 22% probability of negative ones. By contrast, extreme negative values of SPI (below the 10th percentile) led to the occurrence of negative pointer years at 76% probability.

**Table 2:** **Posterior summary from “***coda***” for tree-ring width (W_**r**_) and specific hydraulic conductivity (K_**s**_) bayesian logistic regression fit**.

	**W_r_**		**K_s_**	
	**(Intercept)**	**β_SPI_**	**(Intercept)**	**β_SPI_**
*Mean*	0.002	0.092	0.049	0.98
SD	0.20	0.21	0.20	0.22
SE	0.0009	0.0010	0.0007	0.0008
2.5%	−0.400	−0.323	−0.345	0.548
25%	−0.135	−0.051	−0.080	0.824
50%	0.002	0.091	0.049	1.974
75%	0.140	0.234	0.184	1.129
97.5%	0.405	0.517	0.446	1.444

*Marginal posterior distribution of mean, standard deviation, and quantiles for α (intercept) and β_SPI_. SPI, Standardized Precipitation Index. Trace and density plots are on Supplementary Figures S1, S2*.

## Discussion

Notable negative relationships were detected in Figure 1 between radial growth and vessel traits; we can therefore reasonably rule out the hypothesis of a direct growth-dependent constraint on the intra-annual xylem hydraulic traits. In former studies, this inverse relationship was reported when comparing wood anatomy of both ring porous (Phelps and Workman, 1994; Fonti and García-González, 2004; Gea-Izquierdo et al., 2012) and diffuse porous hardwood species (Denne et al., 1999; Rita et al., 2015). On the other hand, according to the recent tendencies, we cannot exclude an age-dependent constraint on the whole-plant hydraulic function (Olson et al., 2014; see Section Discussion below).

As a whole, there are a number of interesting considerations that can be drawn from our GAM model. First, our findings reported a discernible climatic signal of K_s_ compared to W_r_ (Table 1 and Figure 2), in accordance with many recent studies on the Mediterranean ring-porous (García-González and Eckstein, 2003; Campelo et al., 2010), diffuse-porous (Rita et al., 2015), and conifer trees (Olano et al., 2012). This marked link reflects the ability of trees to adjust the characteristics of their xylem hydraulic architecture, such as the arrangement, frequency, and diameter of vessels to climate variability (Hacke et al., 2006; Sperry et al., 2008), and can provide information about the plasticity of a species under changing environmental conditions. For instance, functional relationships between xylem traits of *Q. canariensis* trees growing in the Mediterranean drought-prone sites exhibit both spatial and temporal plasticity in relation to climatic variability (Gea-Izquierdo et al., 2012).

As for the effects of climate on functional anatomical traits, GAMs results confirm the broadly described influence of climatic factors on the variations in wood traits structure. Accordingly, the positive influence of precipitation and the negative effect of high temperature on the specific hydraulic conductivity (K_s_) are considered key features of most Mediterranean tree species (Table 1 and Figure 2). Indeed, rise in temperature and reduced water availability, that concomitantly lead to an intensification in evapotranspiration, are often been reported to strongly reduce the vessel lumen area and increase their density in order to reduce vulnerability to embolism (Lo Gullo et al., 1995; Tyree and Zimmermann, 2002). Accordingly, many valuable results from long-term time series of xylem traits of sub-Mediterranean oaks emphasized greater phenotypic plasticity in response to the stressful climate conditions (González-González et al., 2014 among the others). In particular, the earlywood vessels lumen area of *Q. robur* were found to decrease in response to reduced spring rainfall (García-González and Eckstein, 2003); similar findings were also highlighted by for *Q. ilex* (Campelo et al., 2010). Further recent evidences showed correlations between earlywood vessels size and precipitation along the previous growing season for *Q. petraea*, whereas the number of vessels was related to winter temperature for the sub-Mediterranean *Q. pyrenaica* (González-González et al., 2014).

In our study, relationships with cambial age over time underlined a strong ontogenetic influence on both growth and specific hydraulic conductivity (Figures 2, 3). In this regard, consistent with the pattern found by other authors for ring-porous *Quercus* spp. (e.g., Heliñska-Raczkowska, 1994; Lei et al., 1996; Fonti and García-González, 2004; Leal et al., 2006), most of the variability in vessel size might be explained by cambial age. Their results showed an overall increasing trend in vessel size and a slight tendency for the conductive area to increase with cambial age. Such a relationship can be largely explained by functional reasons. For instance, multiple lines of evidence suggest that ontogenetic changes in wood anatomy have evolved primarily to provide hydraulic safety in long distance water transport (Anfodillo et al., 2006; Preston et al., 2006; Poorter et al., 2010). Therefore, ontogenetic trends are known to reflect an adaptive compromise between growth constraints and the environment, which is why they should be carefully modeled and interpreted, rather than routinely removed by means of standardization procedures (Carrer et al., 2015). Indeed, the age-specific tree-ring data analyses of Voelker (2011) showed that the sensitivity of tree growth to environmental variability changes predictably with tree age and that the growth of older forests may be more resilient to climate change effects.

Despite the long-standing recognition of the importance of climate extremes on the overall functional performance of trees, the study of climate extremes is a relatively new emphasis in ecology (Smith, 2011). In this case, one advantage of Bayesian methods was the ability to directly answer specific questions in terms of probability of success. In this study we found no direct-related effect of the SPI on tree-ring growth (W_r_) departures, according to the role of “compensatory process” argued by Lloret et al. (2012). This result is also supported by a short-term manipulative experiment on three young deciduous oaks species exposed to artificial air warming and drought: monitored growth reaction showed that, despite a phenological shift induced by warming, annual growth and shoot biomass were not affected by the exposed drought (Kuster et al., 2014).

On the other hand, the probability of extreme departure in specific hydraulic conductivity (K_s_) rises at extreme values of SPI (Figure 5). What emerged in our study provides new insights into the effect of severe climate events on K_s_ from long-term tree-ring series. Indeed, while a generic link between pointer years (*sensu* Schweingruber et al., 1990) and peculiar climate occurrences are fully investigated (Rolland et al., 2000; Neuwirth et al., 2007; Rita et al., 2014), no specific temporal pattern between climate extremes and functional traits was well addressed. Therefore, by relying on our results, we believe that the occurrence of extreme SPI (below the 10th percentile) may lead to an adverse effect on the xylem water transport capability for this species at the study site. In fact, although the xylem structure can acclimate to variation during growth and development by plastically adjusting its xylem anatomical traits (Fonti et al., 2010), the presence of extreme climate events may undermine this anatomical adaptation strategy leading to embolism and related dysfunctions (Choat et al., 2012; Urli et al., 2013). In this regard, there is much experimental evidences suggesting that extreme drought stress is a trigger factor inducing hydraulic failure in trees, resulting in loss of carbon assimilation rate (Brodribb et al., 2010; Urli et al., 2013), shoot dieback (Hoffmann et al., 2011), and tree mortality (Carnicer et al., 2011; Barigah et al., 2013). In particular, our results are consistent with Fonti et al. (2013) which document a significant vessel size reduction with diminished conductivity in saplings of three oak species artificially drought-exposed over three consecutive growing seasons. Moreover, physiological measurements conducted by Nardini et al. (2013) highlighted diffuse crown desiccation in *Q. pubescens* trees caused by hydraulic failure during an extreme drought. Therefore, assuming departures in K_s_ during extreme events in the current climate, it is conceivable that increased frequency or magnitude of extreme climate events with more adverse conditions would lead to higher reduction of K_s_ and greater incidence of xylem dysfunctions. Thus, the intensification in local extremes rather than average climatic conditions might affect woody plant survival.

## Conclusions

In this paper we sought to describe the most likely effect of frequency of extreme climate events on tree hydraulic system capacity. By relying on our results, we pointed out several important aspects of how climate variability can affect xylem function of two Mediterranean oak species. Thus, our main results may be summarized as follows:

– This study demonstrates the usefulness of long-term xylem trait chronologies as records of environmental conditions at annual resolution. In turn, we highlight that vessel traits prove to have a better climatic signal than tree-ring width.
– Most of the variability in tree-ring width and specific hydraulic conductivity might be explained by cambial age. In modeling and interpreting long-term time-series of xylem anatomical features, ontogenetic trends should not be overlooked or ruled out, but carefully evaluated based on every climatic and environmental growth constraint.
– Local extremes are closely linked to the specific hydraulic conductivity in two Mediterranean oak species. Therefore, changing frequency or intensity of extreme events might overcome the adaptive limits of vascular transport, resulting in substantial reduction of hydraulic functionality, and hence increased incidence of xylem dysfunctions.

## Author contributions

AR conceived and designed the study; carried out the measurements; performed analysis of data; wrote the manuscript; AS and LT collected plant materials and contributed to the chronology building; AR, MB, LT, AS contributed to discussing and interpreting the data at all stages.

### Conflict of interest statement

The authors declare that the research was conducted in the absence of any commercial or financial relationships that could be construed as a potential conflict of interest.
