# Supplementary material for: Interpreting the Climatic Effects on Xylem Functional Traits in Two Mediterranean Oak Species: The Role of Extreme Climatic Events
Source: Front Plant Sci. 2016 Aug 2;7:1126. doi: 10.3389/fpls.2016.01126 (PMC4970489; doi:10.3389/fpls.2016.01126)
Supplement: Supplementary file 1 [file Image1.PDF]

Supplementary Material

Interpreting the climatic effects on xylem functional traits in two Mediterranean oak species: the role of extreme climatic events

Angelo Rita[1,2,*], Marco Borghetti[1], Luigi Todaro[1], Antonio Saracino[2]

[1] *Scuola di Scienze Agrarie, Forestali, Alimentari e Ambientali, Università della Basilicata, Viale dell'Ateneo Lucano 10, 85100 Potenza, Italy.*

[2] *Dipartimento di Agraria, Università di Napoli Federico II, Via Università 100, 80055 Portici, Italy.*

*** Correspondence:** Angelo Rita, Dipartimento di Agraria, Università di Napoli "Federico II", Via Università 100, 80055 Portici, Italy.
mail: *angelo.rita@unina.it;* phone: +39 081 2539389

1 Supplementary Figures and Tables

1.1 Supplementary Figures

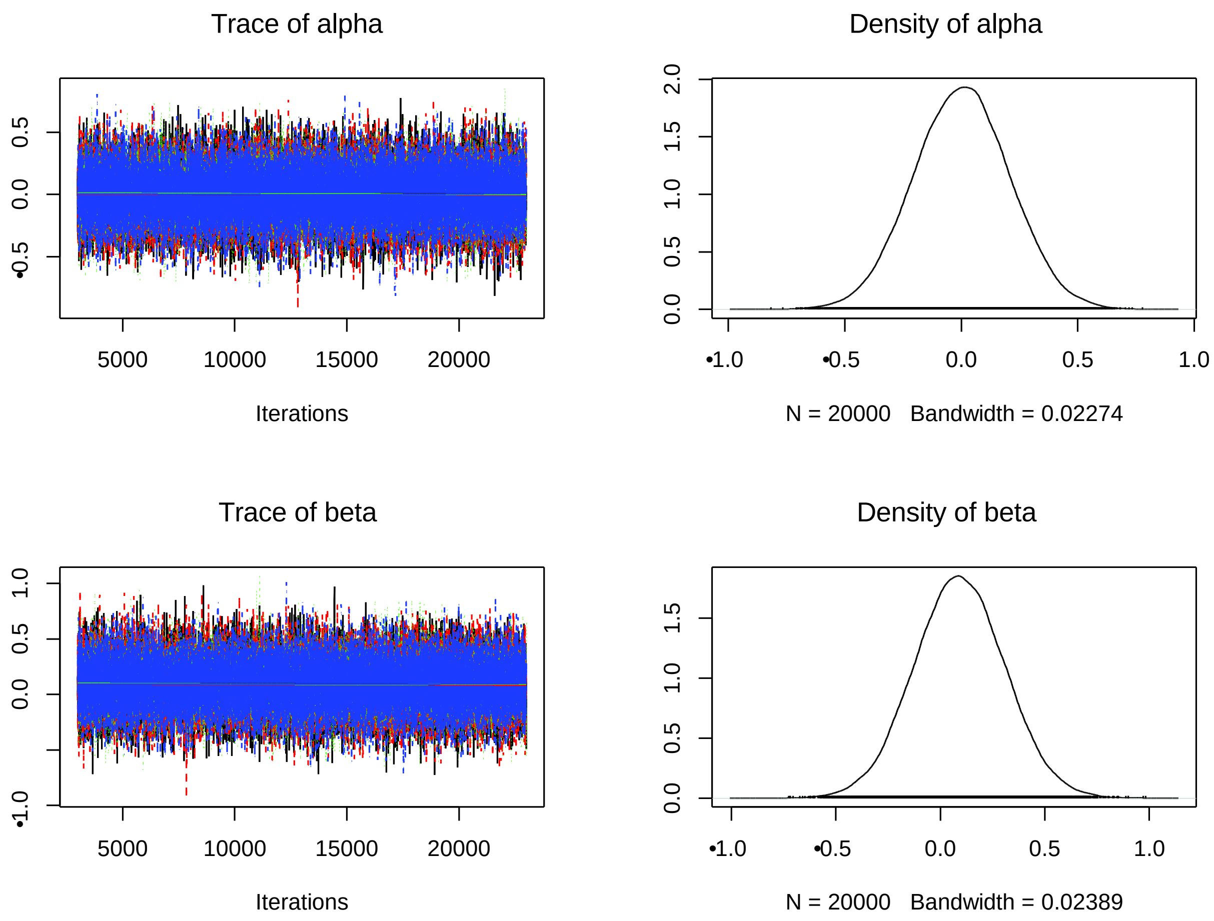

Supplementary Figure S1: Wr. Trace (left side) and density plots (right side) for the posterior sample. Colors in the trace of coefficients versus the iteration of MCMC represent the individual chains.

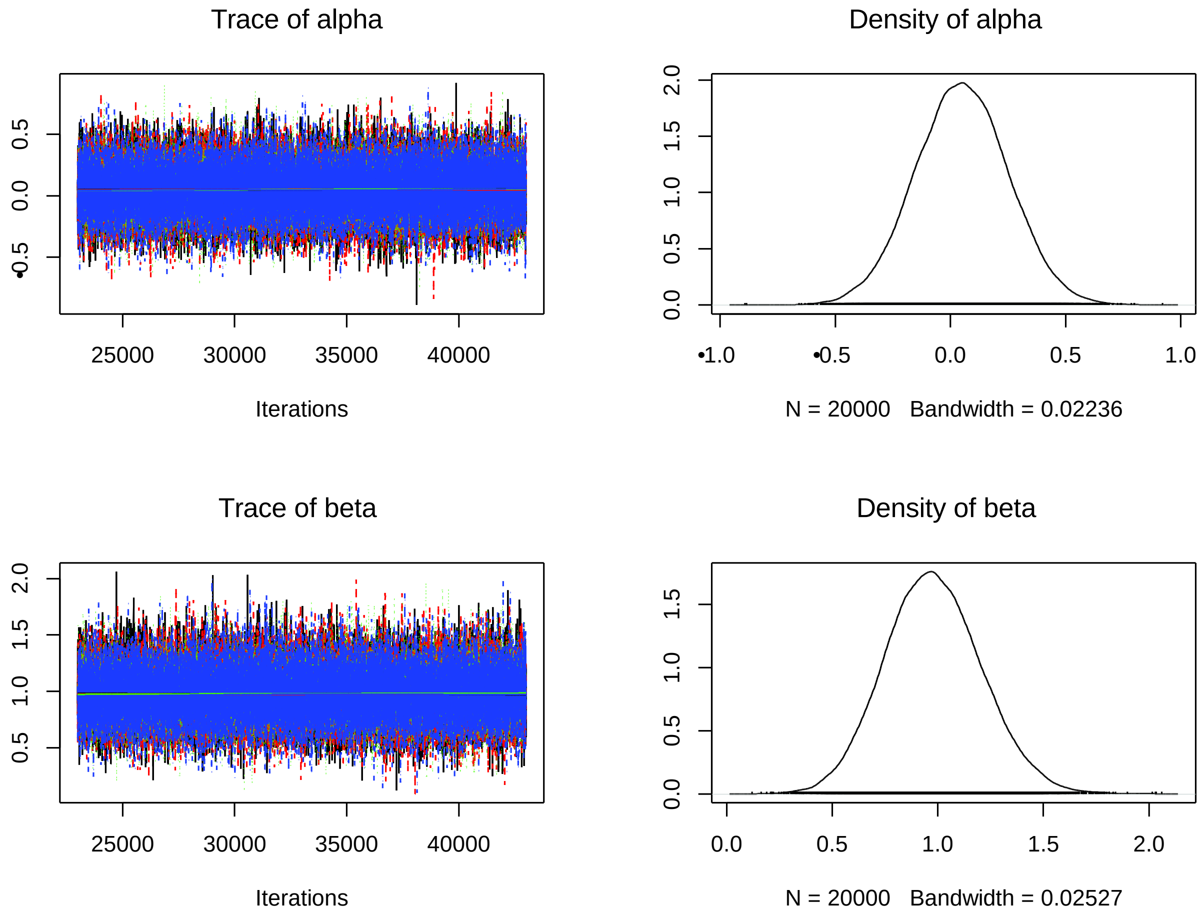

Supplementary Figure S2: Ks. Trace (left side) and density plots (right side) for the posterior sample. Colors in the trace of coefficients versus the iteration of MCMC represent the individual chains.
